# Effect of epoxy value on the rheological properties and microcosmic mechanism of WER emulsified asphalt

**DOI:** 10.1371/journal.pone.0296202

**Published:** 2024-01-26

**Authors:** Dongqing Huang, Junyi Shi, Zongyan Ouyang

**Affiliations:** 1 Guangzhou City Center Transportation Project Management Center, Guangzhou, China; 2 College of Transportation Engineering, Tongji University, Shanghai, China; 3 The Key Laboratory of Road and Traffic Engineering, Ministry of Education, College of Transportation Engineering, Tongji University, Shanghai, China; 4 Southwest Jiaotong University and Leeds Joint School, Sichuan, China; Shandong University of Technology, CHINA

## Abstract

Waterborne epoxy resin (WER), a cleaning material with exceptional high-temperature resistance, has attracted much attention to modify emulsified asphalt in the pavement material field. Epoxy value is the critical characteristic index of WER. In this research, three WER with the epoxy values of 0.20 eq/100g, 0.44 eq/100g, and 0.51 eq/100g were utilized as asphalt modifiers. The influence of epoxy value on WER-EA was investigated by comparing the rheological properties of three kinds of WER emulsified asphalt (WER-EA). The modification mechanism of WER-EA has been analyzed using FTIR and SEM. The results demonstrate that different WER-EA resulted in significantly different rheological properties. WER-EA with the epoxy value of 0.20 eq/100g (E20) performed best at high temperatures, with a maximum increase of 17477% in G*/sin*δ* compared to the neat asphalt and a maximum increase of 66.3% in G*/sin*δ* compared to the other two WER-EA. WER-EA with 0.44 eq/100g epoxy value (E44) performed best at low temperatures, with a maximum increase in *m* value of 39.4% and a maximum decrease in *S* value of 33.3% compared to the other two WER-EA. In addition, the interpenetrating polymer network (IPN) in E20 was observed to be more solid and stable, and IPN in E44 was more uniform. To summarize, lower epoxy value led to a higher degree of WER reaction and higher content of rigid groups, which is more conducive to optimizing the high-temperature property of WER-EA. WER with moderate epoxy value resulted in a low content of polar bonds and thus high content of flexible segments, which helps emulsified asphalt to form a more uniform IPN.

## Introduction

Waterborne epoxy resin emulsified asphalt (WER-EA) is one type of high-performance asphalt material prepared by adding WER and the corresponding curing agent to emulsified asphalt [1]. Waterborne epoxy resin (WER) has the same excellent performance as epoxy resin while being more environmentally friendly [2–4]. At present, bisphenol-A WER is the most commonly used type of emulsified asphalt modifier.

**Fig 1:**
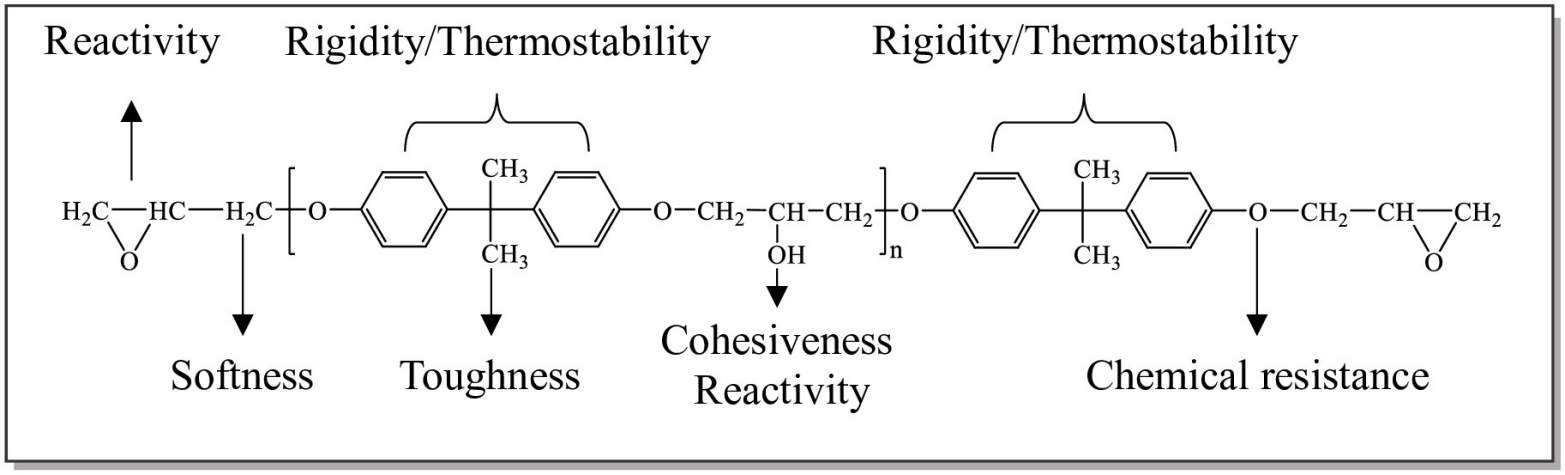
Different properties of different functional groups of WER.

**Fig 2:**
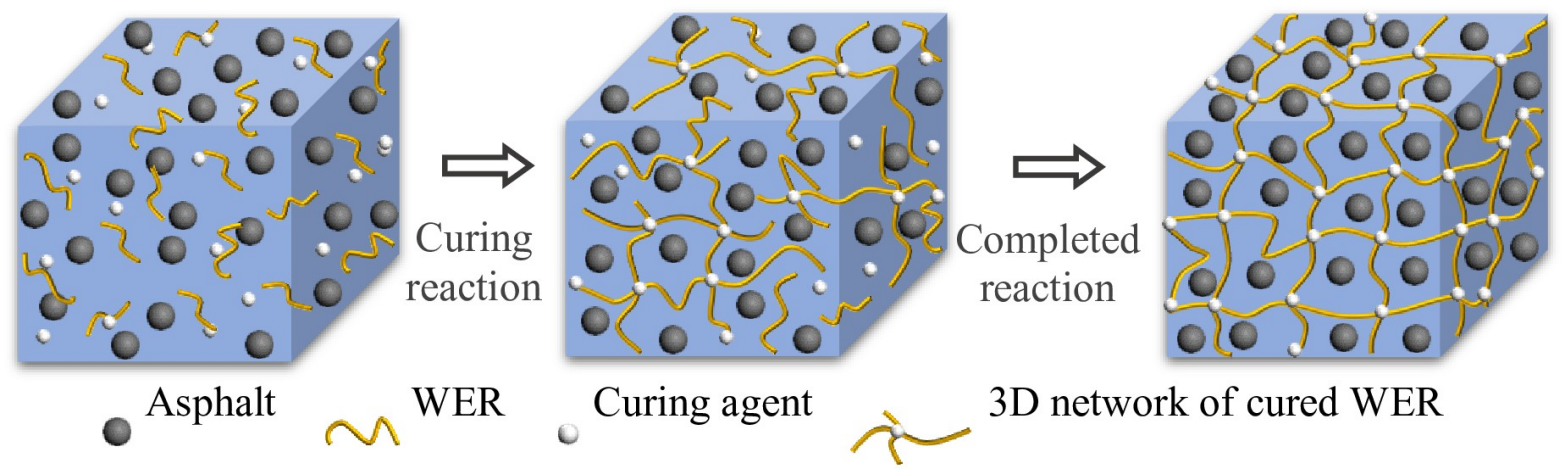
The reaction of the WER system in emulsified asphalt.

In the molecular structure of WER, different functional groups give WER different properties [5] (Fig 1). Epoxy group endows the WER with reactivity so that the cured WER has strong cohesion and adhesion [6]. The benzene ring gives WER stiffness. In addition, the longer the molecular chain, the higher the flexibility of WER. After adding the amine curing agent to WER-EA [7], the epoxy group polymerizes with the amine group of the curing agent to form a high molecular weight polymer [8, 9]. And then, the high molecular weight polymer disperses in emulsified asphalt to form the interpenetrating polymer network (IPN) [10], which will become more complete as the curing reaction progresses [11, 12] (Fig 2). IPN is very stable under high temperature and pressure. Therefore, WER-EA has higher strength, thermal stability, and durability [13].

Epoxy value $E_{v}=2\times \frac{100}{M}$ (M: Molecular weight of epoxy resin) is the main index of WER, representing the epoxy groups’ content in epoxy resin molecules and the degree of WER reaction. Because the strength is related to the degree of crosslinking, cured WER with high epoxy value has higher strength but greater brittleness. On the contrary, cured WER with low epoxy value have insufficient strength and better flexibility at high temperatures [14, 15]. The epoxy value directly determines the performance of WER, so there will be apparent differences in the influence of different epoxy values on WER-EA. However, users usually do not understand the differences between these modifiers [16, 17], which leads to differences in the evaluation of the modification effect of WER on emulsified asphalt. Yu et al. believed that when WER dosage was more than 30%, the continuous network structure was formed to optimize the property of emulsified asphalt [18]. Meltem found that at a 2% WER dosage, the rheological property of WER-EA was the best [8]. G*/sin*δ* of WER-EA prepared by Li et al. was significantly higher than that of Wang et al. at the exact dosage. In general, the research on WER-EA has always been a concern [19]. Yin et al. optimized the performance of WER with an epoxy value of 0.2 eq/100g emulsified asphalt to make it suitable for micro-surfacing [20]. Yan et al. used WER with an epoxy value of 0.44 eq/100g to study the rheological properties of emulsified asphalt [21]. Sun et al. reviewed the laboratory research on the skid resistance of asphalt pavement modified by WER with the epoxy value of 0.51 eq/100g and nano-silica composite [22]. Nevertheless, a single kind of WER-EA is usually used in previous studies, resulting in insufficient research on the differences in the effects of epoxy values on WER-EA.

To analyze the modification characteristics of epoxy value on WER-EA, three kinds of bisphenol-A WER with different epoxy values were selected in this study. The three WER adopted are mature in preparation technology, widely used and lower in cost. Their epoxy values were 0.20 eq/100g, 0.44 eq/100g, and 0.51 eq/100g, respectively. Emulsified asphalt was modified with five WER dosages (0%, 5%, 10%, 15%, and 20%) and their evaporation residues was prepared. The physical performance of WER-EA evaporation residues was analyzed through three main indicators. The rheological behavior of WER-EA evaporation residues was analyzed through temperature sweep (TS), multiple stress creep recovery (MSCR), and bending beam rheometer (BBR) tests. Finally, the modification mechanism was explained by Fourier transform infrared reflection (FTIR) and scanning electron microscopy (SEM) tests. This study can provide a reference for selecting WER with corresponding epoxy values according to performance requirements.

## Materials and experiment

### Emulsified asphalt

The cationic emulsified asphalt prepared from 70# neat asphalt was selected. The fundamental properties of emulsified asphalt are shown in Table 1.

**Table 1:** Fundamental properties of emulsified asphalt.

Property		Results	JTG E20-2019
Particle charge		+	+
1.18-mm screen residues (%)		0.04	≤0.1
Solid content (%)		63	≥55
Evaporation residue	Penetration (25°C, 0.1mm)	64.2	45–150
	Softening point (°C)	48.5	-
	Ductility (15°C, cm)	80.3	≥40

### WER

Bisphenol-A WER with various epoxy values was used in this experiment. WER was purchased from ChemChina, and the physical properties are shown in Table 2.

**Table 2:** Performance indicators of three kinds of WER.

Index	Unit	Test result		
		E20	E44	E51
Appearance	—	Milky white uniform liquid		
**Epoxy value**	**eq/100g**	**0.21**	**0.44**	**0.51**
Epoxy equivalent weight	g/mol	456	227	196
Solid content	%	50±2		

### Curing agent

The aliphatic amine curing agent was purchased from ChemChina. The essential performance of the curing agent is shown in Table 3.

**Table 3:** Essential performance of curing agent (C_6_H_18_N_4_).

Index	Appearance	Molecular weight	Amine value	Solid content
Measured values	Light yellow viscous liquid	146.23	1498.0 mgKOH/g	50±2%

### Preparation and test methods

#### Preparation methods

WER-EA samples with different dosages were prepared first. In order to evaluate the performance of samples, their evaporation residues were carried out [19]. Modifier dosage and sample preparation procedure are determined concerning the previous experimental results [23].

1. Put an appropriate amount of emulsified asphalt into the container and mix it with a mixer with a rotating speed of 1000 r/min. Slowly add WER into emulsified asphalt during mixing (the dosages used in this research are 5%, 10%, 15%, and 20% of the mass fraction of emulsified asphalt, respectively). Stir for 5min after WER is added. After thoroughly mixing the emulsified asphalt and WER, add the curing agent, which is 20% of the mass fraction of WER, to the mixture at a uniform speed. WER-EA can be prepared by stirring the mixture for 10 minutes.
2. Heat 200-300g of WER-EA in an electric furnace with a surface temperature between 200°C and 250°C. Stir manually at an average speed (200-300rpm) until the temperature of the evaporated residues reaches 160°C. Place the product in a 163°C oven until it has no bubbles. The insulation process shall be controlled within 5 minutes. The temperature should be well controlled in the preparation process. Otherwise, the product shall fail due to agglomeration [24].

#### Test methods

**Fig 3:**
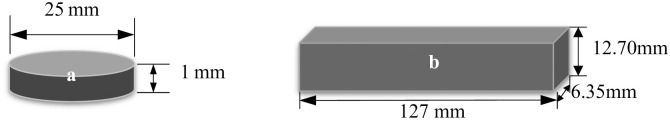
Sample sizes of TS/MSCR test (a) and BBR test (b).

**Fig 4:**
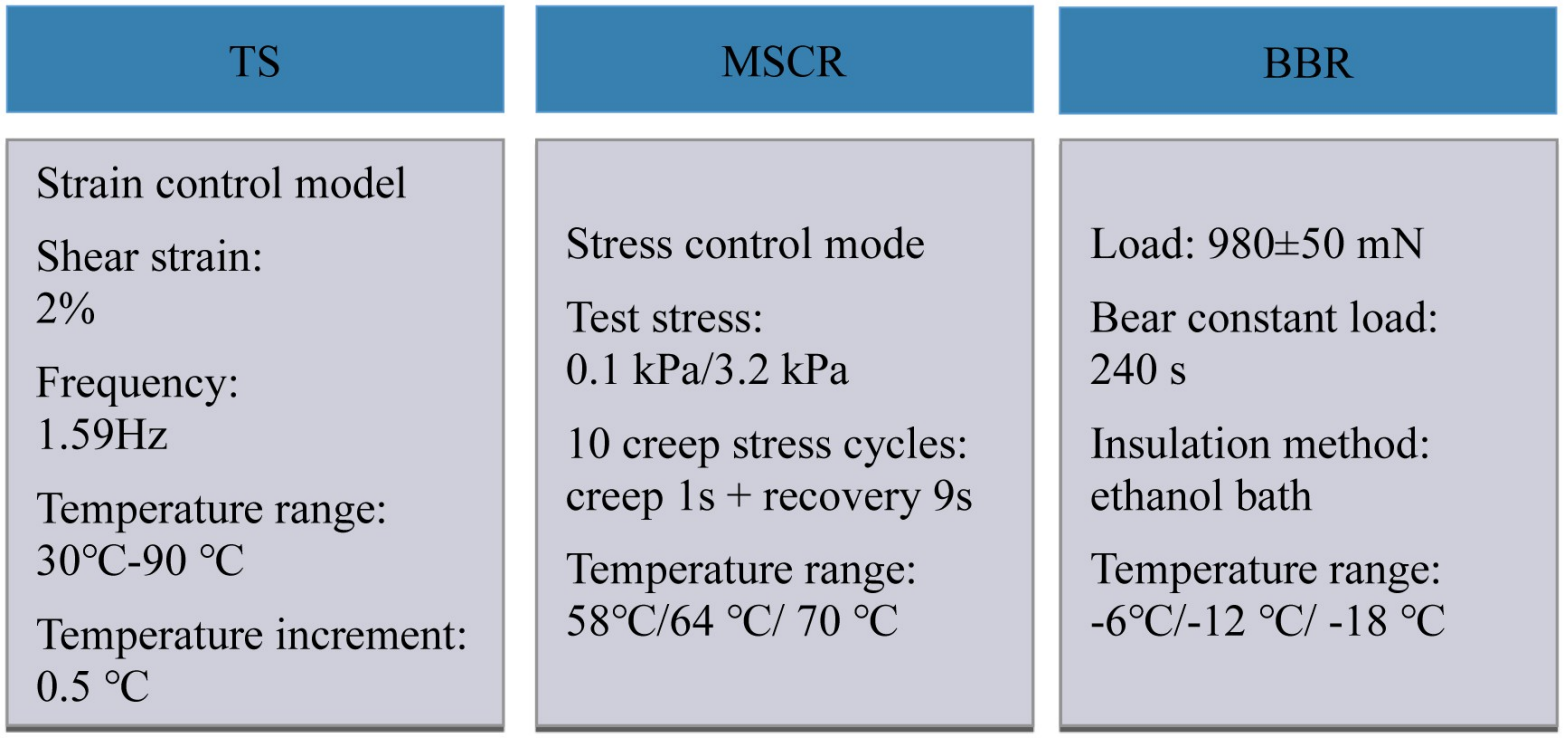
Test procedures of rheological property.

The physical properties and rheological properties tests in this study were conducted following JTG E20-2011, and each index was measured by averaging from three groups of parallel tests. TS, MSCR, and BBR tests are utilized to test the high- and low-temperature rheological properties of WER-EA. Fig 3 shows the sizes of the samples. Test procedures for rheological property test are shown in Fig 4 [6, 25–29].

FTIR and SEM tests are utilized to reveal the microcosmic mechanism of WER-EA [30]. The spectral range of FTIR was 500 cm^-1^-4000cm^-1^, the scanning frequency was 20 times/min, and the resolution was 4 cm^-1^ [31, 32]. The magnification (Mag) of SEM images tested at 5 kV voltage is 50.00 KX [33].

## Results and discussion

### Physical properties

**Fig 5:**
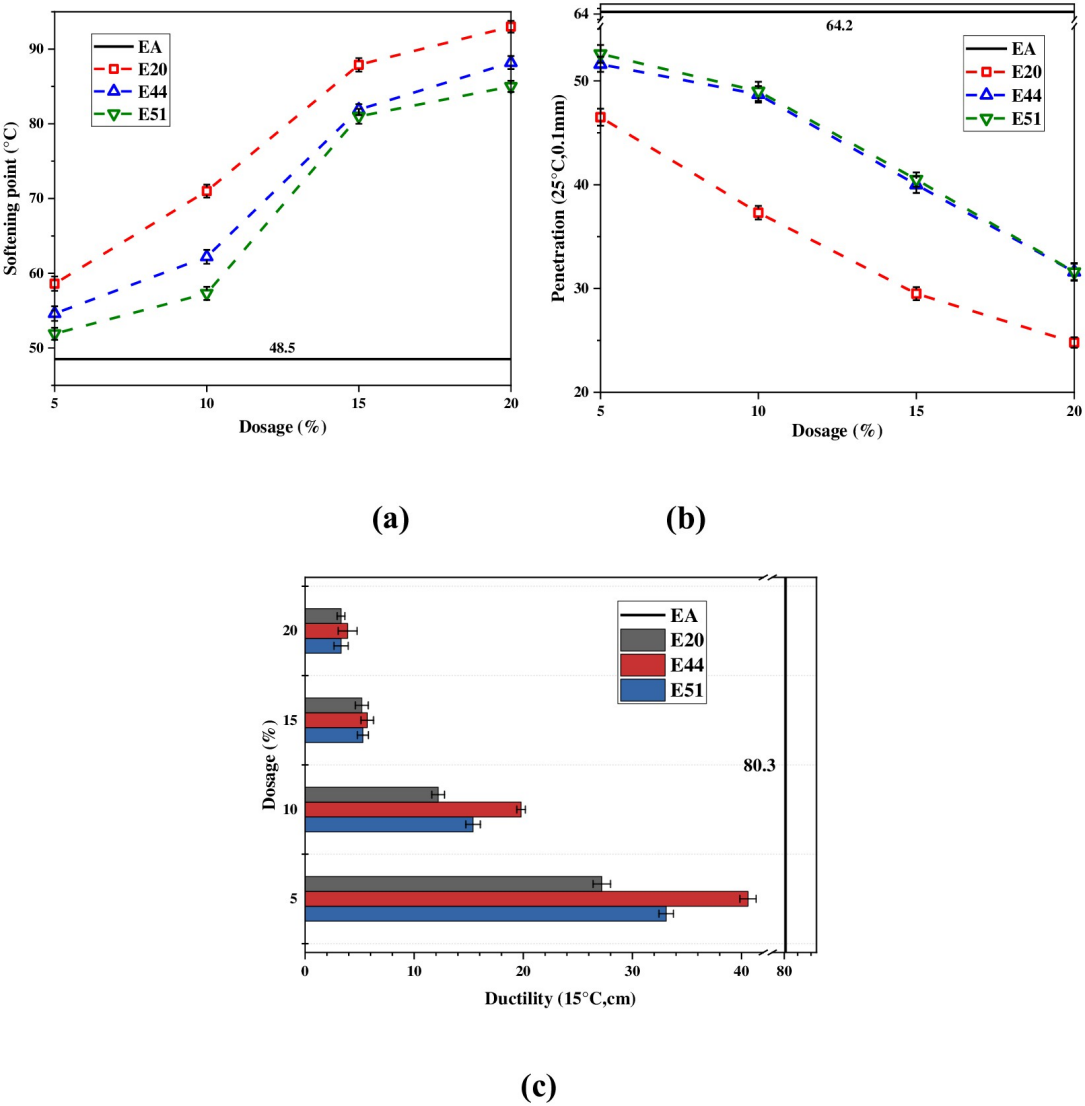
Physical properties of three WER-EA.

Penetration reflects the viscosity of asphalt. Softening point reflects the viscosity and thermal stability of asphalt. Ductility is the plasticity index of asphalt. In Fig 5, EA represents emulsified asphalt, while E20, E44, and E51 represent emulsified asphalt modified by WER with epoxy values of 0.2 eq/100g, 0.44 eq/100g, and 0.51 eq/100, respectively.

When the dosage of three WER increases from 0% to 20%, the penetration and ductility of E20/E44/E51 decrease, and the softening point of E20/E44/E51 increases. Fig 5(a) and 5(b) demonstrates that when the dosage is 5%, 10%, 15%, and 20%, the penetration and softening point of E44 and E51 are similar, but the penetration of E44 is slightly lower, and the softening point of E44 is slightly higher. The minimum penetrations of E20/E44/E51 evaporation residues are 23.8 (0.1mm), 31.6 (0.1mm), and 31.6 (0.1mm), respectively, which decrease by 61.4% (E20), 50.8% (E44) and 50.8% (E51), respectively compared with EA. Compared with EA, the maximum softening points of WER-EA increase by 91.8% (E20), 81.9% (E44), and 75.3% (E51), respectively. This is consistent with previous research findings that the addition of WER can significantly improve the high-temperature performance of emulsified asphalt. Fig 5(c) demonstrates that the ductility has the following numerical relationship under the same conditions: E44 > E51 > E20. However, when WER dosage is 15% or 20%, the ductility is numerically similar. When WER dosage is close to 20%, the ductility and penetration of WER-EA are close to the limit values. Therefore, this study agrees with the conclusion of Zhang et al. that it is recommended to control WER dosage within 20% [3].

Fig 5 suggests that E20 has better high-temperature and thermal stability, while E44 has better low-temperature performance than the other two evaporation residues. These conclusions are consistent with the later rheological experimental findings.

### Temperature sweep test

**Fig 6:**
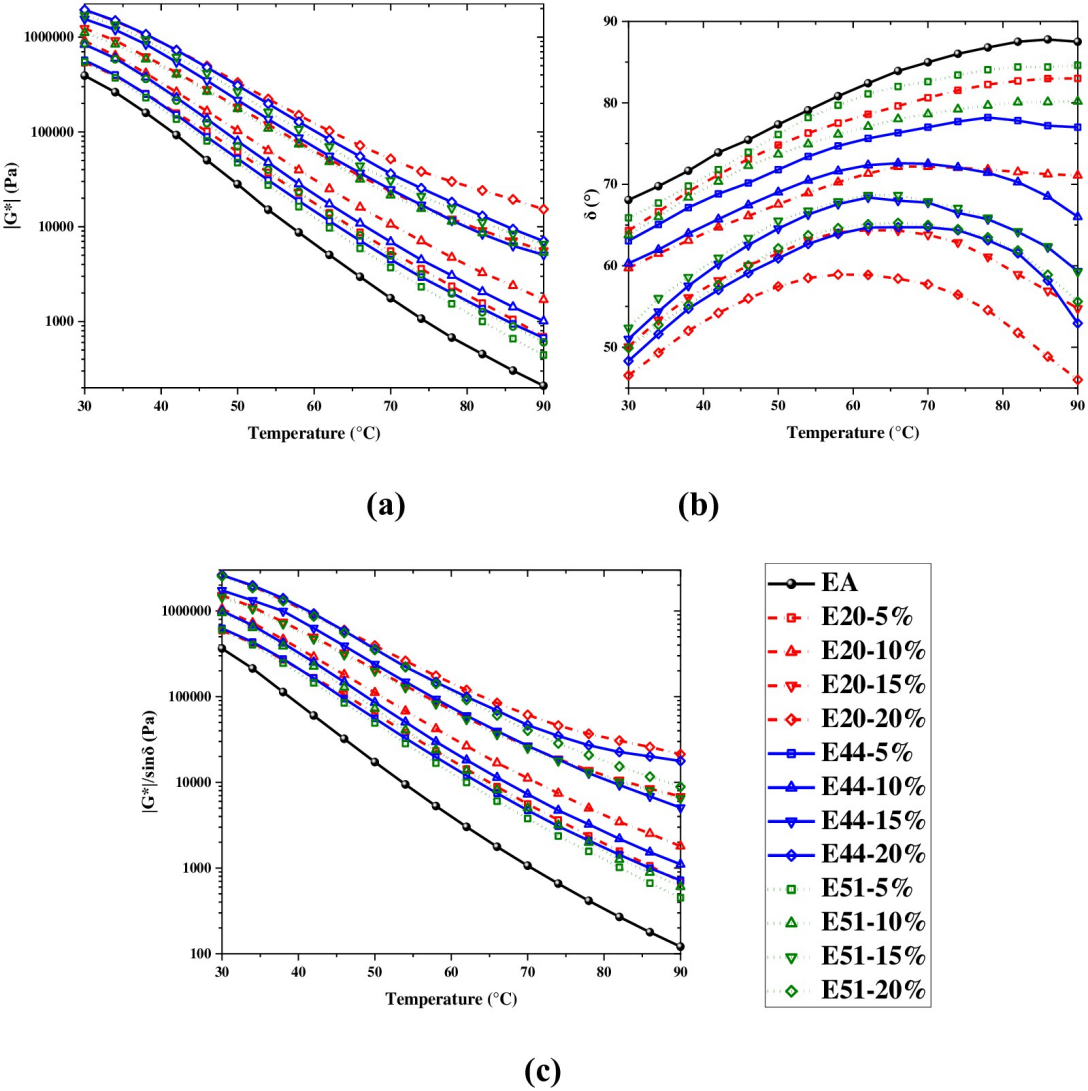
G*, *δ* and G*/sin*δ* at different temperatures.

The G*, *δ*, and G*/sin*δ* of samples are obtained through temperature sweep (TS) tests to characterize their deformation resistance and viscoelasticity at high temperatures. Fig 6 demonstrates that G*, *δ*, and G*/sin*δ* of samples have the same change rules with the dosage increase. The reason may be that IPN produced in WER-EA optimizes its high-temperature performance. The *δ* of WER-EA has a peak value because WER makes emulsified asphalt have thermosetting characteristics [19].

When the dosages are 5%, 10%, and 20%, there is an obvious observation of G*: G*_E20_ > G*_E44_ > G*_E51_, and the law becomes more pronounced as temperature rise. Between 30°C and 90°C, the G* of E20 and E44 is larger than that of E51. When WER dosage is 15% and the test temperature is less than 66°C, the G* of E44 is significantly greater than that of E20 and E51, while the G* of E20 is slightly greater than that of E51. When WER dosage is 15% and the test temperature is 66°C-90°C, the three G* are similar. The relationship of the *δ* is regular: when WER dosage is 5%, *δ*_*E51*_>*δ*_*E20*_>*δ*_*E44*_; when WER dosage is 10%, the *δ* of E20 is obviously smaller than that of E20 and E51 while the *δ* of E20 and E51 is similar; when WER dosage is 15% or 20%, the *δ* of E20 is smaller than that of E44 and E51 while the *δ* of E44 is slightly smaller than that of E51. The quantitative relationship of G*/sin*δ* of the three materials is consistent with that of G*: at the dosages of 5%, 10%, and 20% G*/sin*δ*_E20_ > G*/sin*δ*_E44_ > G*/sin*δ*_E51_, while the values of G*/sin*δ* are similar at 15% dosage. Compared with research results of Wang et al., WER-EA in this experiment shows a 17328% increase in G*/sin*δ* under the same conditions, resulting in better high-temperature performance [19].

The characteristics of functional groups, the crosslinking structure of polymers, and the motion characteristics of chain segments are the main reasons for the performance differences of WER emulsified asphalt. What’s more, when the epoxy value is 0.44 eq/100g or 0.51 eq/100g, the average degree of polymerization (*n*) of WER is 0 ~ 1; when the epoxy value is 0.2 eq/100g, the *n* of WER is 1.8–5. Fig 1 indicates that the greater the *n*, the greater the proportion of the benzene ring in WER. Benzene ring, which can optimize the thermal stability and rigidity of WER-EA, is a group with solid rigidity. In addition, Chen et al. found that in addition to the type of WER, the amount of curing agent also affected the reaction degree of WER, thereby affecting the performance of WER cured products [2]. In the test, a unified mass of curing agent was used, which was 20% of WER. However, the optimal theoretical amine mass consumption of E20, E44, and E51 are 9.75%, 21.43%, and 24.86% of WER, respectively. So, the epoxy group reaction degree of E20 is greater, the crosslinking degree is higher, and the formed skeleton structure makes the asphalt have strong resistance to deformation when heated. On the contrary, there are unreacted polar groups in E44 and E51, which leads to an increase in matrix brittleness and lower mechanical properties compared to E20. Therefore, WER with an epoxy value of 0.2 eq/100g has a better effect on WER-EA performance under high temperatures.

### MSCR test

**Fig 7:**
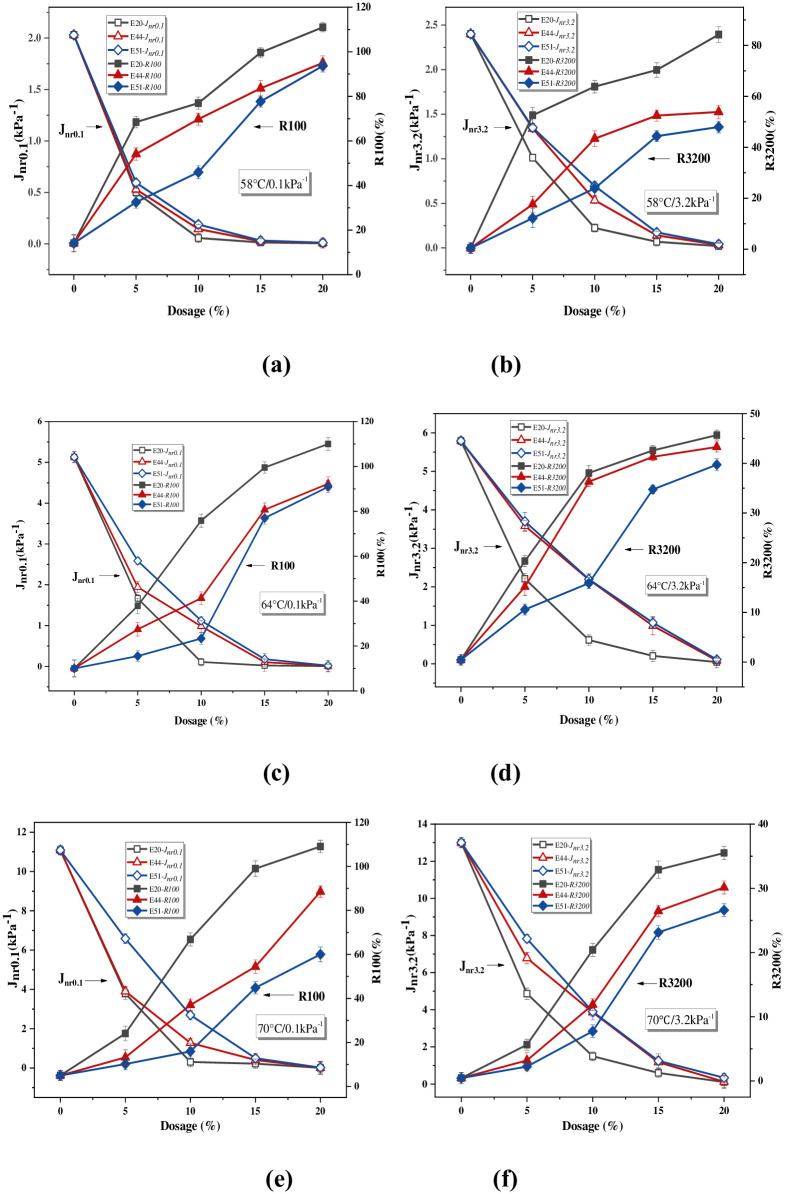
*J*_*nr*_ and *R* of different samples.

*J*_*nr*_ and *R* obtained by MSCR indicate the permanent deformation resistance of WER-EA [34]. Whether under high pressure (3.2kPa) or low pressure (0.1kPa), low *J*_*nr*_ or high *R* means that WER-EA has strong deformation resistance. Fig 7 shows WER can optimize the deformation resistance of emulsified asphalt, and the improvement effect becomes evident with the increase of WER content.

The trend of changes in low-temperature rheological indicators in this study is consistent with the trend studied by Li et al [14]. Under the same conditions, the three modifiers have obvious differences in improving the performance of emulsified asphalt. When the experimental temperatures are 58°C, 64°C and 70°C, the *J*_*nr0*.*1*_ and *J*_*nr3*.*2*_ of samples show the same rule: *J*_*nr*_ (E20) < *J*_*nr*_ (E44) < *J*_*nr*_ (E51). In addition, *J*_*nr*_ (E20) is significantly less than *J*_*nr*_ (E44) and *J*_*nr*_ (E51), while *J*_*nr*_ (E44) is close to *J*_*nr*_ (E51) (especially when the pressure is 3.2 kPa). Under the three temperature conditions used in the test, *R* under stresses of 0.1kPa and 3.2kPa shows the same law: *R*(E20) >*R*(E44) >*R*(E51).

There is a significant difference in the *R* and *J*_*nr*_ of samples, which shows that epoxy values apparently influence the viscoelastic of WER-EA. E20 has better deformation resistance because it has lower *J*_*nr*_ and higher *R*. This is due to the higher degree of epoxy group reaction in E20 and the high proportion of ether bond (R-O-R’) and carbon-carbon single bonds (C-C), which increases the flexibility and movement ability of molecular chain of E20. In addition, the IPN in E20 has a significant degree of interpenetrating entanglement. When stressed, it can produce the relative slip of molecular chain segments like linear molecules, which is conducive to the elastic-plastic deformation of the material.

### BBR test

**Fig 8:**
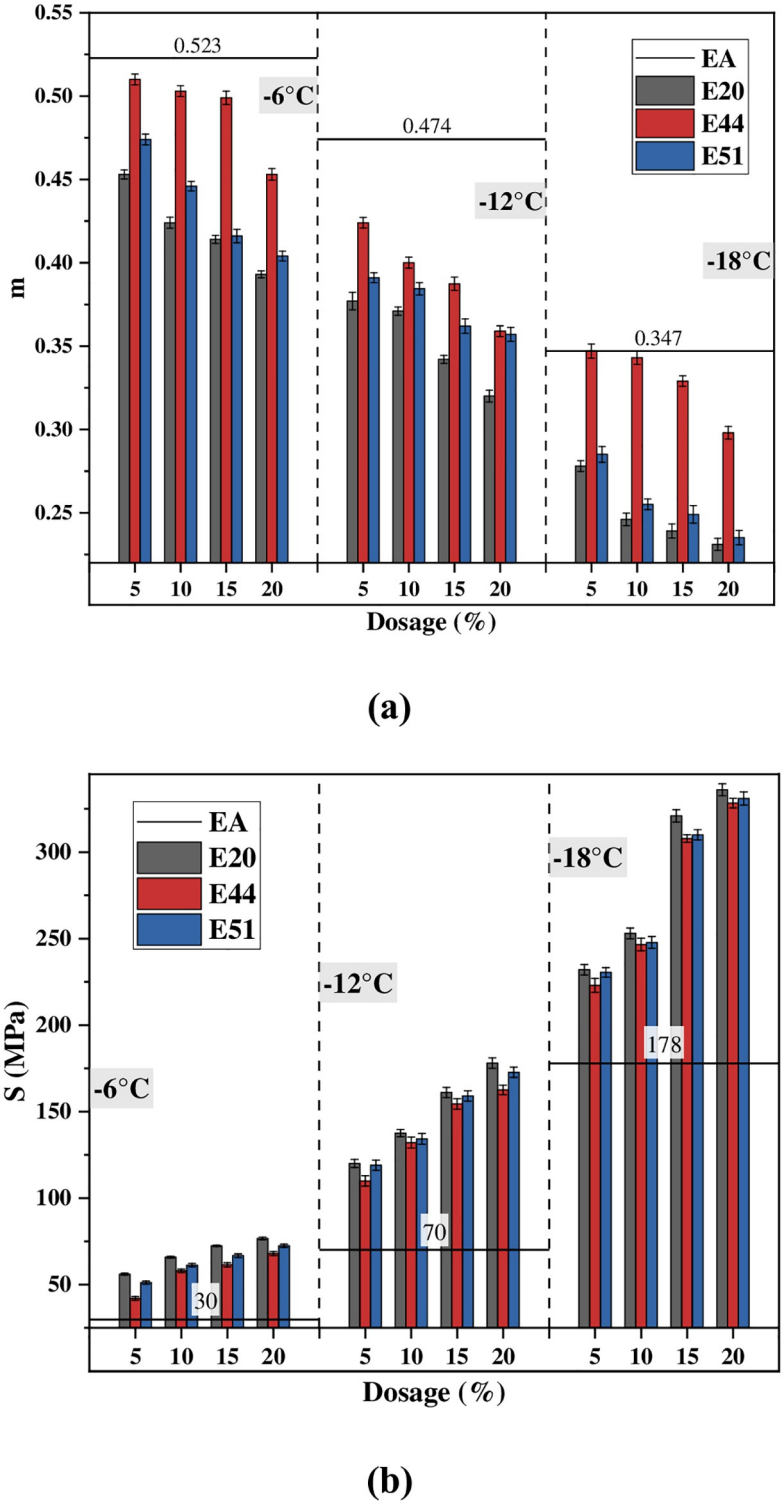
*m* and *S* at different temperatures and dosages.

Two indexes, flexural stiffness modulus (*S*) and creep rate (*m*), are obtained by the BBR test to evaluate the low-temperature performance of WER-EA evaporation residues. Fig 8 indicates that WER weakens the low-temperature performance of emulsified asphalt. At a temperature of -18°C, E20 and E51 are unsuitable for use (*m* less than 0.3 on Superpave) due to the benzene ring’s brittleness in WER affecting the ductility of asphalt at low temperatures. This also shows that WER-emulsified asphalt is more suitable for tropical and subtropical regions.

Different WER weakens the low-temperature performance of emulsified asphalt differently. Similar to the research conclusion of Wang et al., when WER dosage reaches 10%, the possibility of low-temperature performance of WER-EA not meeting the specifications is greater [19]. When the experimental temperatures are -6°C, -12°C, and -18°C, *m* (E44) is significantly greater than *m* (E51) and *m* (E20), and *m* (E51) is slightly greater than *m* (E20). Under the same experimental conditions, *S*(E44) < *S*(E51) < *S*(E20). However, when the temperature decreases from -12ºC to -18ºC, the difference of *S* of the three evaporation residues becomes smaller. The results show that WER, with an epoxy value of 0.44 eq/100g, has the least adverse effect on emulsified asphalt. This is due to the high proportion of benzene rings and polymer segments in E20. The synergistic effect of the benzene ring increases the material’s brittleness, the mobility of polymer segments is poor, and the steric effect is strong.

E44 and E51 have a higher content of flexible segments. However, there are more polar bonds in the crosslinking structure of E51, which makes it more brittle. Therefore, controlling the epoxy value of WER within a specific range can make WER-EA have ideal mechanical properties and little adverse impact on toughness.

### FTIR test

**Fig 9:**
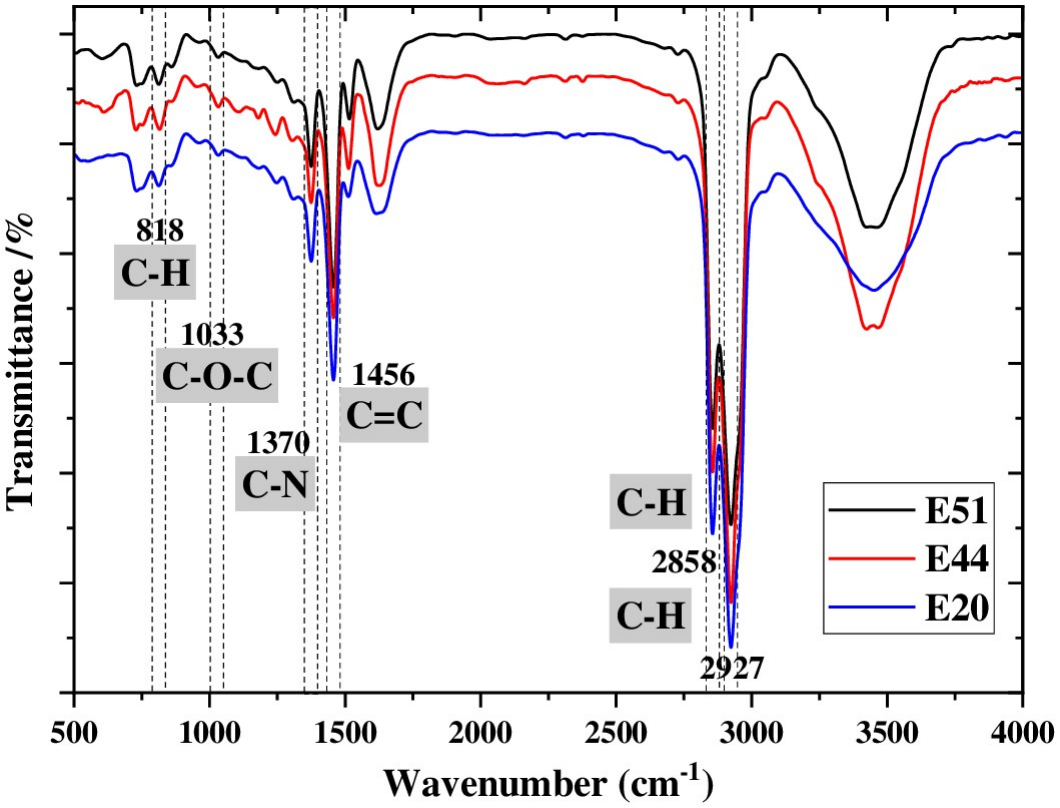
FTIR at 20% dosage.

According to the rheological test analysis, it has been found that the change in the performance of WER-EA was due to the effect of different internal functional groups, and the functional groups of the sample with the highest dosage (20%) used in this study has the most pronounced effect. So, the functional groups in the sample with the dosage of 20% were analyzed. Fig 9 demonstrates that the 818 cm^-1^ peak signifies the out-of-plane bending vibration of C-H, which represents a para-disubstituted benzene ring. The peak of C-O-C at 1033 cm^-1^ shows the stretching asymmetric stretching vibration of alicyclic ether and the symmetric stretching vibration of aryl ether. The peak at 1377 cm^-1^ signifies the C-N of the tertiary amine. The peak at 1456 cm^-1^ represents C = C, indicating the stretching vibration of the benzene ring. The peaks at 2858 cm^-1^ and 2927 cm^-1^ signify the symmetric stretching vibration and asymmetric stretching vibration of C-H on alkanes, respectively.

The peaks at 818, 1033, and 1456 cm^-1^ are the characteristic peaks of WER in emulsified asphalt. Only tertiary amine exists in WER-EA, which indicates that the added curing agent (C_6_H_18_N_4_) has wholly reacted to form the interpenetrating polymer network. The results verify that some specific functional groups in WER-EA can affect its performance, which is consistent with the above mechanism analysis.

### SEM

**Fig 10:**
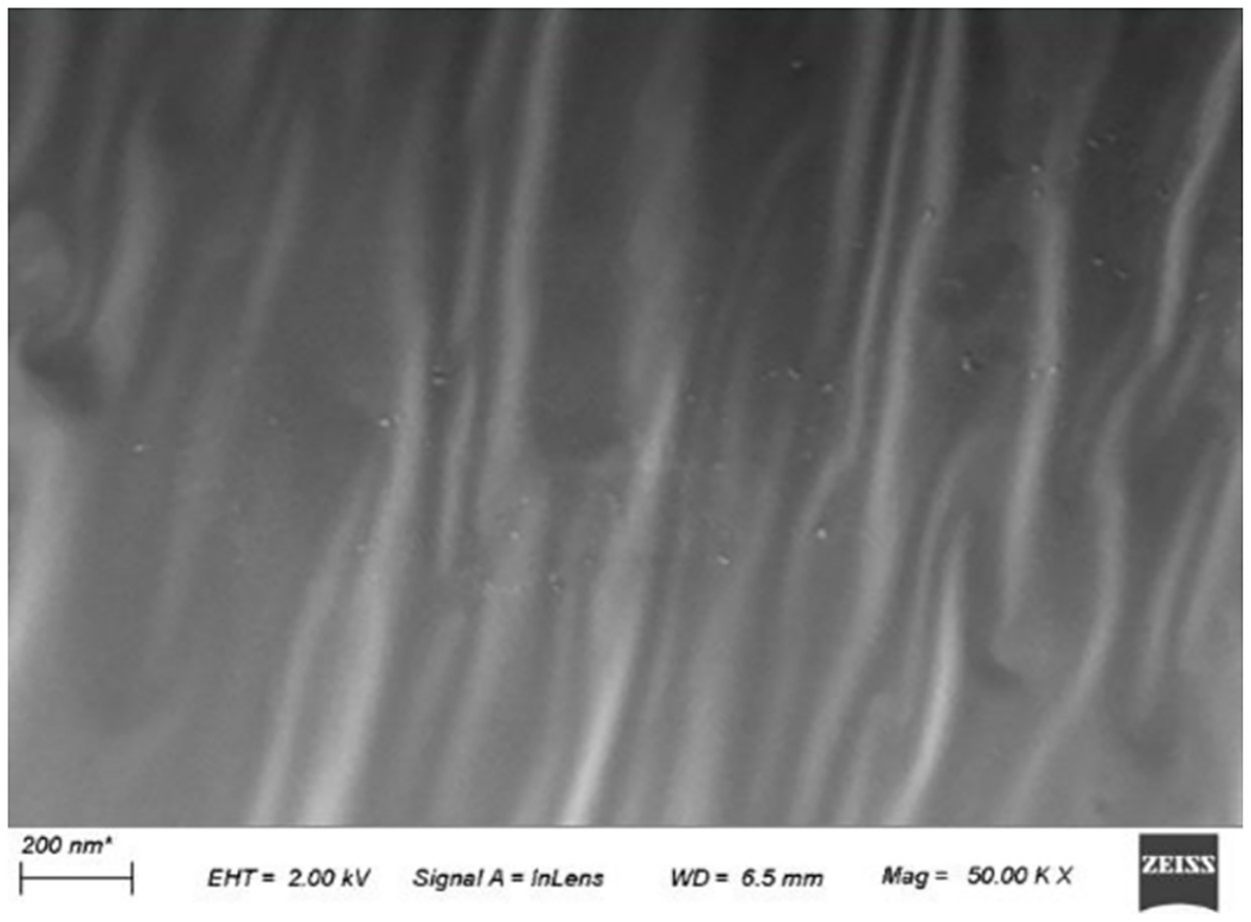
SEM images of emulsified asphalt evaporation residues.

**Fig 11:**
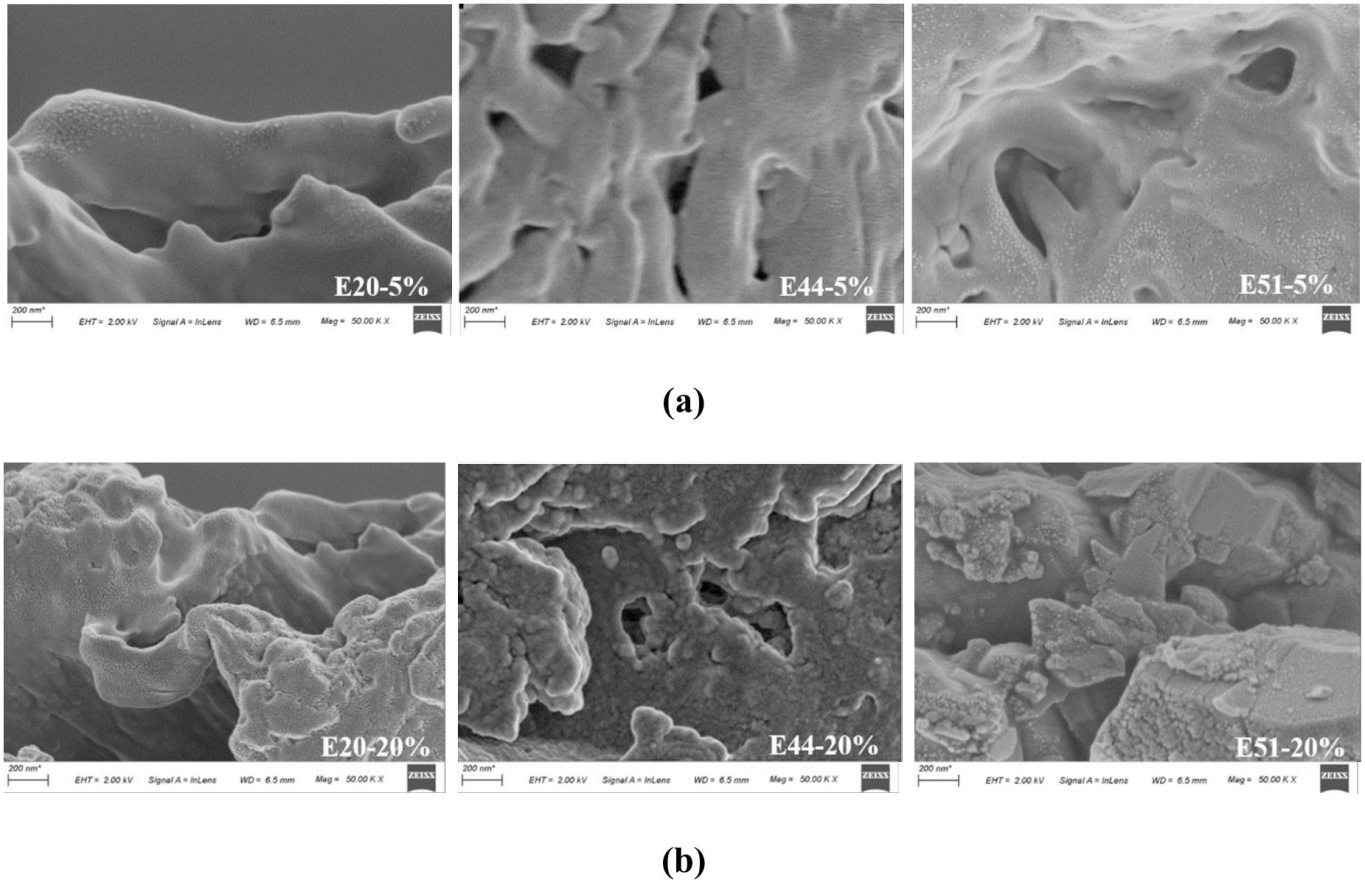
SEM images of WER-EA evaporation residues.

Fig 10 shows the SEM image of emulsified asphalt evaporated residue. Fig 11 shows the SEM images of WER-EA evaporated residues when the WER system dosage is 5% (the lowest dosage) or 20% (the highest dosage).

Compared to Fig 10 (only flat folds in the sample), Fig 11 shows an interpenetrating polymer network (IPN) in WER-EA, which is why its performance is different from that of pure emulsified asphalt. Fig 11 demonstrates that the crosslinking degree of IPN increases as the dosage increases, making the effect of IPN more evident and the influence range of IPN larger. Overall, the images obtained in this experiment are consistent with existing images, showing a three-dimensional skeleton structure in WER-EA [8, 19]. However, the skeleton structure shown in Fig 3 is more pronounced. Compared with E44 and E51, the quality of IPN formed in E20 is higher. IPN of E20 has a broader influence range, higher integrity, more robust connection, etc. In addition, IPN in E44 evaporation residue is more uniform.

There are many factors affecting the IPN of WER-EA evaporation residues. However, the main influencing factors are the degree of interpenetration, crosslinking, and network composition. The higher the epoxy group reaction degree of WER, the higher the degree of interpenetration and cross-linking. However, too much crosslinking will be detrimental to the toughness of the cured product. The flexible chain segment in WER can be bonded to the dense epoxy resin crosslinking network and IPN, resulting in microscopic phase separation and forming a thick and loose two-phase network structure, which improves the property of WER-EA. E20 has a higher degree of crosslinking, and E44 has a higher content of flexible segments. Hence the skeleton structure in the E20 evaporation residue is more solid and stable, and the skeleton structure in the E44 evaporation residue is more uniform. However, under the same test conditions, the content of unreacted groups in E51 is higher, reducing the degree of microphase separation and producing uneven phase structure. Hence, E20 has the best high-temperature performance, while WER, with the epoxy value of 0.20 eq/100g, has the most negligible impact on emulsified asphalt.

## Conclusion

To analyze the modification characteristics of epoxy value on WER-EA, three different WER with epoxy values of 0.2 eq/100g, 0.44 eq/100g, and 0.51 eq/100g were utilized to prepare WER-EA. Through the analysis of rheological behavior and microstructure, four conclusions are summarized.

1. The influence of WER on emulsified asphalt is positively correlated with the dosage, and the maximum effect of dosage is 20%. Adding WER with the epoxy value of 0.20 eq/100g resulted in the most remarkable change in the penetration/softening point. With the epoxy value of 0.44 eq/100g, WER has the least adverse effect on the ductility.
2. WER optimizes the high-temperature property of emulsified asphalt. The temperature sweep test shows that E20 has better high-temperature performance, with a maximum increase of 66.3% in G*/sin*δ* compared to the other two WER-EA. MSCR test shows that E20 has better deformation resistance. The lower the epoxy value, the higher the content of the rigid group, and the more conducive to optimizing the high-temperature properties of WER emulsified asphalt.
3. WER has an adverse impact on the low-temperature performance of WER-EA. WER with the epoxy value of 0.44 eq/100g has the least negative effect on WER-EA, with a maximum increase in *m* value of 39.4% and a maximum decrease in *S* value of 33.3% compared to the other two WER. This is because WER with moderate epoxy value has a high content of flexible segments and a low content of polar bonds, which makes a less adverse impact on the toughness of WER-EA.
4. Overall, the performance of E51 is not superior to that of E20 and E44, but it is far superior to that of neat asphalt. It is worth noting that E51 can show better performance under certain conditions. For example, when WER dosage is 15%, the difference between G*/sin*δ* of E51 and E20 is only 5%, and the difference between *S* of E51 and E44 is only 0.71%. This provides a reference for the application of WER-EA under certain conditions.
5. The FTIR test verifies that some specific functional groups in WER-EA can affect its performance. SEM images show that WER with low epoxy value can make IPN in WER-EA more stable and stable, while WER with medium epoxy value can make IPN in WER-EA more uniform.

In general, WER emulsified asphalt is more suitable for hot areas and roads with significant traffic volume. The simple preparation process and high performance make it can be used as paving material and cold patching material. WER with the epoxy value of 0.2 eq/100g has the best high-temperature performance, and its application can improve road performance compared with other WER. In the future, the performance of WER with an epoxy value of 0.2 eq/100g asphalt mixture should be characterized to comprehensively evaluate its application’s value.

## Supporting information

S1 File (DOCX)
