# Supplementary material for: Effect of epoxy value on the rheological properties and microcosmic mechanism of WER emulsified asphalt
Source: PLoS One. 2024 Jan 26;19(1):e0296202. doi: 10.1371/journal.pone.0296202 (PMC10817193; doi:10.1371/journal.pone.0296202)
Supplement: S1 File — (DOCX) [file pone.0296202.s001.docx]

Physical properties

| Penetration(25°C,0.1mm) |  |  |  |
| --- | --- | --- | --- |
| Dosage(%) | E20 | E44 | E51 |
| 0 | 64.2 | 64.2 | 64.2 |
| 5 | 46.5 | 52.6 | 0.72807 |
| 10 | 37.3 | 49 | 0.79257 |
| 15 | 29.5 | 40.5 | 0.79485 |
| 20 | 24.8 | 31.6 | 0.8519 |

| Softening point(°C) |  |  |  |
| --- | --- | --- | --- |
| Dosage(%) | E20 | E44 | E51 |
| 0 | 48.5 | 48.5 | 48.5 |
| 5 | 58.6 | 54.6 | 51.9 |
| 10 | 71 | 62.2 | 57.3 |
| 15 | 87.9 | 81.9 | 81 |
| 20 | 93 | 88.2 | 85 |

| Ductility(15°C,cm) |  |  |  |
| --- | --- | --- | --- |
| Dosage(%) | E20 | E44 | E51 |
| 0 | 80.3 | 80.3 | 80.3 |
| 5 | 33.1 | 40.6 | 27.2 |
| 10 | 15.4 | 19.8 | 12.2 |
| 15 | 5.3 | 5.7 | 5.2 |
| 20 | 3.3 | 3.9 | 3.3 |

Temperature sweep test

| Temperature | \|G*\| | \|G*\| | \|G*\| | \|G*\| | \|G*\| | \|G*\| | \|G*\| | \|G*\| | \|G*\| | \|G*\| | \|G*\| | \|G*\| | \|G*\| |
| --- | --- | --- | --- | --- | --- | --- | --- | --- | --- | --- | --- | --- | --- |
| ℃ | Pa | Pa | Pa | Pa | Pa | Pa | Pa | Pa | Pa | Pa | Pa | Pa | Pa |
|  | EA | E20-5% | E20-10% | E20-15% | E20-20% | E44-5% | E44-10% | E44-15% | E44-20% | E51-5% | E51-10% | E51-15% | E51-20% |
| 30 | 391590 | 540490 | 910120 | 1234000 | 1918720 | 568760 | 836540 | 1551700 | 1942600 | 549520 | 857130 | 1114600 | 1701700 |
| 34 | 262140 | 393290 | 637740 | 920070 | 1485100 | 398730 | 597990 | 1195200 | 1494400 | 371940 | 584230 | 843400 | 1345200 |
| 38 | 158860 | 252700 | 415500 | 623750 | 1055200 | 250710 | 378260 | 839920 | 1071300 | 229690 | 362850 | 586900 | 939920 |
| 42 | 92701 | 157560 | 262980 | 423100 | 730180 | 151450 | 229960 | 545810 | 728800 | 136700 | 213910 | 405770 | 615810 |
| 46 | 50331 | 101510 | 165460 | 280550 | 496050 | 88440 | 136610 | 347020 | 480580 | 80416 | 123420 | 267730 | 417020 |
| 50 | 28069 | 61709 | 103000 | 183400 | 332230 | 52318 | 79930 | 216360 | 309630 | 47355 | 70496 | 175850 | 266360 |
| 54 | 15096 | 37177 | 63494 | 119730 | 222520 | 30996 | 47634 | 136410 | 198530 | 27431 | 39919 | 109110 | 162410 |
| 58 | 8681.4 | 22673 | 39653 | 77830 | 149880 | 18711 | 28283 | 86688 | 127420 | 16275 | 23509 | 74300 | 106688 |
| 62 | 5035.7 | 13984 | 25088 | 51313 | 102370 | 11475 | 17353 | 55726 | 83094 | 9731 | 13524 | 48540 | 70126 |
| 66 | 2960 | 8718.8 | 16075 | 34319 | 71903 | 7123.7 | 10873 | 36850 | 55022 | 5901.7 | 8103.6 | 31756 | 43850 |
| 70 | 1761.3 | 5514.5 | 10618 | 23662 | 51887 | 4514.8 | 6936.5 | 24692 | 36223 | 3710.4 | 4982.2 | 21473 | 30692 |
| 74 | 1070.9 | 3593.8 | 7075.6 | 16672 | 38276 | 2939.1 | 4476.3 | 17003 | 25475 | 2323.3 | 3148.7 | 15533 | 21003 |
| 78 | 676.57 | 2352.1 | 4743.9 | 12073 | 29963 | 1998.3 | 3061.9 | 11652 | 18171 | 1538.1 | 1973.8 | 11401 | 15652 |
| 82 | 453.2 | 1562.4 | 3274.9 | 9115 | 24086 | 1361.4 | 2061.5 | 8376.4 | 12995 | 1000.7 | 1251.6 | 8605 | 11176.4 |
| 86 | 303.22 | 1050.8 | 2387.4 | 7090.7 | 19407 | 943.28 | 1422.5 | 6281.2 | 9424.5 | 657.26 | 884.43 | 6786 | 8381.2 |
| 90 | 209.96 | 689.82 | 1701.1 | 5579.9 | 15301 | 670.75 | 1011.3 | 5050.5 | 7048.7 | 443.58 | 602.65 | 5350 | 6547.5 |

| Temperature | δ | δ | δ | δ | δ | δ | δ | δ | δ | δ | δ | δ | δ |
| --- | --- | --- | --- | --- | --- | --- | --- | --- | --- | --- | --- | --- | --- |
| ℃ | ° | ° | ° | ° | ° | ° | ° | ° | ° | ° | ° | ° | ° |
|  | EA | E20-5% | E20-10% | E20-15% | E20-20% | E44-5% | E44-10% | E44-15% | E44-20% | E51-5% | E51-10% | E51-15% | E51-20% |
| 30 | 68.08 | 64.32 | 59.73 | 50.08 | 46.54 | 63.06 | 60.3 | 51.01 | 48.31 | 65.89 | 63.78 | 52.37 | 49.91 |
| 34 | 69.77 | 66.62 | 61.49 | 53.35 | 49.32 | 65.06 | 61.97 | 54.37 | 51.63 | 67.7 | 66 | 56.01 | 52.75 |
| 38 | 71.66 | 69.13 | 63.1 | 56.1 | 52.02 | 67.14 | 63.95 | 57.54 | 54.73 | 69.78 | 68.4 | 58.6 | 55.2 |
| 42 | 73.9 | 71.17 | 64.74 | 58.2 | 54.2 | 68.83 | 65.71 | 60.21 | 57.04 | 71.8 | 70.34 | 60.97 | 57.65 |
| 46 | 75.45 | 73.11 | 66.11 | 60.04 | 55.99 | 70.16 | 67.45 | 62.54 | 59.12 | 73.93 | 72.28 | 63.4 | 60.03 |
| 50 | 77.34 | 74.81 | 67.54 | 61.53 | 57.46 | 71.8 | 69.03 | 64.56 | 60.9 | 76.12 | 73.68 | 65.51 | 62.14 |
| 54 | 79.09 | 76.29 | 68.93 | 63.15 | 58.5 | 73.41 | 70.47 | 66.29 | 62.68 | 78.19 | 74.94 | 66.75 | 63.75 |
| 58 | 80.82 | 77.52 | 70.26 | 64.11 | 58.93 | 74.7 | 71.63 | 67.57 | 63.92 | 79.71 | 76.13 | 67.9 | 64.61 |
| 62 | 82.4 | 78.59 | 71.33 | 64.37 | 58.89 | 75.63 | 72.35 | 68.38 | 64.69 | 81.1 | 77.09 | 68.67 | 65.09 |
| 66 | 83.91 | 79.61 | 72.17 | 64.33 | 58.42 | 76.32 | 72.59 | 67.99 | 64.73 | 82.01 | 78.02 | 68.66 | 65.26 |
| 70 | 84.98 | 80.63 | 72.17 | 63.8 | 57.72 | 77 | 72.51 | 67.76 | 64.73 | 82.59 | 78.63 | 67.9 | 65 |
| 74 | 86.01 | 81.53 | 72.03 | 62.86 | 56.46 | 77.7 | 72.08 | 66.49 | 64.37 | 83.41 | 79.21 | 67.07 | 64.49 |
| 78 | 86.79 | 82.25 | 71.81 | 61.1 | 54.56 | 78.2 | 71.42 | 65.72 | 63.14 | 84.06 | 79.69 | 65.9 | 63.49 |
| 82 | 87.5 | 82.69 | 71.51 | 58.95 | 51.78 | 77.82 | 70.27 | 64.17 | 61.54 | 84.4 | 80.08 | 64.23 | 61.88 |
| 86 | 87.78 | 82.98 | 71.24 | 56.92 | 48.86 | 77.2 | 68.51 | 62.27 | 58.19 | 84.4 | 80.1 | 62.39 | 58.9 |
| 90 | 87.5 | 83 | 71.09 | 54.76 | 46.01 | 77 | 66.01 | 59.33 | 52.94 | 84.6 | 80.18 | 59.25 | 55.61 |

| Temperature | \|G*\|/sinδ | \|G*\|/sinδ | \|G*\|/sinδ | \|G*\|/sinδ | \|G*\|/sinδ | \|G*\|/sinδ | \|G*\|/sinδ | \|G*\|/sinδ | \|G*\|/sinδ | \|G*\|/sinδ | \|G*\|/sinδ | \|G*\|/sinδ | \|G*\|/sinδ |
| --- | --- | --- | --- | --- | --- | --- | --- | --- | --- | --- | --- | --- | --- |
| ℃ | Pa | Pa | Pa | Pa | Pa | Pa | Pa | Pa | Pa | Pa | Pa | Pa | Pa |
|  | EA | E20-5% | E20-10% | E20-15% | E20-20% | E44-5% | E44-10% | E44-15% | E44-20% | E51-5% | E51-10% | E51-15% | E51-20% |
| 30 | 364500 | 588400 | 1053800 | 1519000 | 2638210 | 625070 | 1003080 | 1734700 | 2615700 | 602570 | 951060 | 1443050 | 2539300 |
| 34 | 212660 | 423700 | 725740 | 1111700 | 1958500 | 433890 | 677480 | 1324200 | 1981300 | 402600 | 636500 | 1056115 | 1852300 |
| 38 | 112910 | 269130 | 465930 | 743000 | 1338700 | 274880 | 421050 | 995460 | 1406400 | 245810 | 388320 | 705850 | 1304700 |
| 42 | 60087 | 166070 | 290780 | 496000 | 900210 | 164270 | 252280 | 628940 | 931740 | 144620 | 225450 | 471200 | 862660 |
| 46 | 32152 | 106080 | 180960 | 324000 | 598440 | 94928 | 147920 | 391070 | 585190 | 84422 | 128440 | 307800 | 554760 |
| 50 | 17249 | 63944 | 111460 | 209000 | 394110 | 55687 | 85600 | 239580 | 359590 | 49343 | 72616 | 198550 | 350230 |
| 54 | 9432.7 | 38267 | 68045 | 134000 | 260960 | 32742 | 50542 | 148990 | 222970 | 28407 | 40783 | 127300 | 221360 |
| 58 | 5279.4 | 23222 | 42128 | 86514 | 174980 | 19670 | 29802 | 93782 | 147390 | 16764 | 23894 | 82188.3 | 141030 |
| 62 | 3018.2 | 14265 | 26482 | 56915 | 119560 | 12020 | 18210 | 59945 | 99636 | 9983.5 | 13689 | 54069.25 | 91614 |
| 66 | 1769.6 | 8864.3 | 16886 | 38078 | 84403 | 7445.5 | 11396 | 39747 | 68823 | 6033.1 | 8183 | 36174.1 | 60581 |
| 70 | 1065.4 | 5589.1 | 11154 | 26372 | 61370 | 4713.1 | 7272.7 | 26677 | 46532 | 3784.6 | 5024.2 | 25053.4 | 40150 |
| 74 | 656.55 | 3633.4 | 7438.5 | 18736 | 45925 | 3069.7 | 4704.6 | 18542 | 34786 | 2365.1 | 3169.7 | 17799.2 | 28468 |
| 78 | 414.69 | 2373.7 | 4993.4 | 13790 | 36779 | 2091 | 3230.3 | 12782 | 27077 | 1563.4 | 1984.4 | 13100.5 | 20799 |
| 82 | 268.9 | 1575.2 | 3453.1 | 10640 | 30659 | 1430.7 | 2190.1 | 9306 | 22611 | 1015.9 | 1257.6 | 10108 | 15343 |
| 86 | 178.28 | 1058.7 | 2521.4 | 8462.8 | 25767 | 998.08 | 1528.8 | 6870.3 | 20044 | 666.15 | 888.98 | 8039.66 | 11665 |
| 90 | 120.99 | 693.08 | 1798.2 | 6832 | 21267 | 718.27 | 1107 | 5058.2 | 17753 | 450.31 | 605.54 | 6490.4 | 8862 |

MSCR test

| 58°C, JNR0.1(kPa-1) | | | |  | 58°C, R100(%) | | | |
| --- | --- | --- | --- | --- | --- | --- | --- | --- |
| Dosage(%) | E20 | E44 | E51 |  | Dosage(%) | E20-R100 | E44-R100 | E51-R100 |
| 0 | 2.03 | 2.03 | 2.03 |  | 0 | 14.1 | 14.1 | 14.1 |
| 5 | 0.5 | 0.529 | 0.59417 |  | 5 | 68.43445 | 54.099 | 32.52127 |
| 10 | 0.05643 | 0.144 | 0.18813 |  | 10 | 76.97912 | 69.765 | 45.96097 |
| 15 | 0.01298 | 0.024 | 0.03145 |  | 15 | 99.64845 | 83.613 | 77.69733 |
| 20 | 0.005 | 0.007 | 0.01 |  | 20 | 111 | 95.033 | 93.67453 |
|  |  |  |  |  |  |  |  |  |
| 58°C, JNR3.2(kPa-1) | | | |  | 58°C, R3200(%) | | | |
| Dosage(%) | E20 | E44 | E51 |  | Dosage(%) | E20-R100 | E44-R100 | E51-R100 |
| 0 | 2.4 | 2.4 | 2.4 |  | 0 | 0.445 | 0.445 | 0.445 |
| 5 | 1.0125 | 1.34 | 1.35 |  | 5 | 52.57988 | 17.605 | 12.20922 |
| 10 | 0.22434 | 0.535 | 0.69532 |  | 10 | 63.82038 | 43.415 | 23.89254 |
| 15 | 0.06927 | 0.141 | 0.17391 |  | 15 | 70.36234 | 52.46 | 44.38652 |
| 20 | 0.02083 | 0.033 | 0.04172 |  | 20 | 84.25512 | 53.879 | 47.96742 |
|  |  |  |  |  |  |  |  |  |
| 64°C, JNR0.1(kPa-1) | | | |  | 64°C, R100(%) | | | |
| Dosage(%) | E20 | E44 | E51 |  | Dosage(%) | E20-R100 | E44-R100 | E51-R100 |
| 0 | 5.129 | 5.129 | 5.129 |  | 0 | 10 | 10 | 10 |
| 5 | 1.66638 | 1.942 | 2.58639 |  | 5 | 37.88616 | 27.426 | 15.52152 |
| 10 | 0.10838 | 0.982 | 1.11936 |  | 10 | 75.86182 | 41.322 | 23.33732 |
| 15 | 0.02163 | 0.104 | 0.178 |  | 15 | 99.50662 | 80.728 | 77.00864 |
| 20 | 0.007 | 0.009 | 0.019 |  | 20 | 110.01842 | 92.274 | 90.91996 |
|  |  |  |  |  |  |  |  |  |
| 64°C, JNR3.2(kPa-1) | | | |  | 64°C, R3200(%) | | | |
| Dosage(%) | E20 | E44 | E51 |  | Dosage(%) | E20-R100 | E44-R100 | E51-R100 |
| 0 | 5.791 | 5.791 | 5.791 |  | 0 | 0.445 | 0.445 | 0.445 |
| 5 | 2.2094 | 3.582 | 3.69987 |  | 5 | 20.32066 | 15.155 | 10.57281 |
| 10 | 0.61812 | 2.188 | 2.19799 |  | 10 | 38.05158 | 36.296 | 15.87296 |
| 15 | 0.20666 | 0.988 | 1.07008 |  | 15 | 42.59244 | 41.304 | 34.74821 |
| 20 | 0.04615 | 0.084 | 0.1119 |  | 20 | 45.67653 | 43.29 | 39.71405 |
|  |  |  |  |  |  |  |  |  |
| 70°C, JNR0.1(kPa-1) | | | |  | 70°C, R100(%) | | | |
| Dosage(%) | E20 | E44 | E51 |  | Dosage(%) | E20-R100 | E44-R100 | E51-R100 |
| 0 | 11.086 | 11.086 | 11.086 |  | 0 | 5 | 5 | 5 |
| 5 | 3.78611 | 3.903 | 6.58739 |  | 5 | 24.08143 | 13.228 | 10.19613 |
| 10 | 0.30875 | 1.278 | 2.69482 |  | 10 | 66.84656 | 37.019 | 15.91893 |
| 15 | 0.223 | 0.411 | 0.5 |  | 15 | 99 | 54.437 | 44.84604 |
| 20 | 0.009 | 0.011 | 0.02 |  | 20 | 109.04528 | 88.548 | 60.03847 |
|  |  |  |  |  |  |  |  |  |
| 70°C, JNR3.2(kPa-1) | | | |  | 70°C, R3200(%) | | | |
| Dosage(%) | E20 | E44 | E51 |  | Dosage(%) | E20-R100 | E44-R100 | E51-R100 |
| 0 | 13.003 | 13.003 | 13.003 |  | 0 | 0.445 | 0.445 | 0.445 |
| 5 | 4.86672 | 6.782 | 7.82633 |  | 5 | 5.645 | 3.208 | 2.24677 |
| 10 | 1.49793 | 3.862 | 3.89828 |  | 10 | 20.41426 | 11.88 | 7.74724 |
| 15 | 0.6 | 1.174 | 1.25361 |  | 15 | 32.9 | 26.467 | 23.13612 |
| 20 | 0.10774 | 0.124 | 0.33762 |  | 20 | 35.5056 | 30.13 | 26.59892 |

BBR test

| m, T=-6°C | | | |  | m, T=-12°C | | | |  | m, T=-18°C | | | |
| --- | --- | --- | --- | --- | --- | --- | --- | --- | --- | --- | --- | --- | --- |
| Dosage(%) | E20 | E44 | E51 |  | Dosage(%) | E20 | E44 | E51 |  | Dosage(%) | E20 | E44 | E51 |
| 0 | 0.523 | 0.523 | 0.523 |  | 0 | 0.474 | 0.474 | 0.474 |  | 0 | 0.347 | 0.347 | 0.347 |
| 5 | 0.453 | 0.51 | 0.474 |  | 5 | 0.377 | 0.424 | 0.391 |  | 5 | 0.278 | 0.347 | 0.285 |
| 10 | 0.424 | 0.503 | 0.446 |  | 10 | 0.371 | 0.4 | 0.3844 |  | 10 | 0.246 | 0.343 | 0.255 |
| 15 | 0.414 | 0.499 | 0.416 |  | 15 | 0.342 | 0.3874 | 0.362 |  | 15 | 0.239 | 0.329 | 0.249 |
| 20 | 0.393 | 0.453 | 0.404 |  | 20 | 0.32 | 0.359 | 0.357 |  | 20 | 0.231 | 0.298 | 0.235 |
|  |  |  |  |  |  |  |  |  |  |  |  |  |  |
| S(MPa), T=-6°C | | | |  | S(MPa), T=-12°C | | | |  | S(MPa), T=-18°C | | | |
| Dosage(%) | E20 | E44 | E51 |  | Dosage(%) | E20 | E44 | E51 |  | Dosage(%) | E20 | E44 | E51 |
| 0 | 30 | 30 | 30 |  | 0 | 70 | 70 | 70 |  | 0 | 187 | 187 | 187 |
| 5 | 56 | 42 | 51.1 |  | 5 | 120 | 109.9 | 119 |  | 5 | 232 | 223 | 230.5 |
| 10 | 65.8 | 57.9 | 61.2 |  | 10 | 137.5 | 132 | 134.2 |  | 10 | 253 | 246.6 | 247.8 |
| 15 | 72.4 | 61.4 | 66.7 |  | 15 | 161 | 154.4 | 159 |  | 15 | 321 | 307.8 | 310 |
| 20 | 76.6 | 67.9 | 72.4 |  | 20 | 178 | 162.5 | 172.7 |  | 20 | 336 | 328.3 | 331 |
|  |  |  |  |  |  |  |  |  |  |  |  |  |  |
